# In Situ Endoscopic Analysis of Vascular Supply and Regenerated Alveolar Bone in *β*-TCP Grafted and Ungrafted Postextraction Sites before Implant Placement: A Prospective Case Control Study

**DOI:** 10.1155/2019/2797210

**Published:** 2019-11-06

**Authors:** Víctor Beltrán, Marcio Lazzarini, Rodolfo Figueroa, Vanessa Sousa, Wilfried Engelke

**Affiliations:** ^1^Universidad de La Frontera, Dental School, Clinical Investigation and Dental Innovation Center (CIDIC) and Center for Translational Medicine (CEMT-BIOREN), Temuco, Chile; ^2^Max Planck Institute of Experimental Medicine, Department of Molecular Biology of Neuronal Signals, Göttingen, Germany; ^3^Universidad de La Frontera, Center of Physics and Engineering in Medicine (CFIM), Faculty of Engineering and Sciences, Temuco, Chile; ^4^Centre for Oral Clinical Research, Institute of Dentistry, Barts & The London School of Medicine & Dentistry, QMUL, London, UK

## Abstract

**Background:**

Endoscopy has seen a significant development over recent years in various medical fields with its application expanding from the support of minimal invasive surgery to in situ imaging. In this context, the application of endoscopic techniques to assess the quality of the regenerated bone in situ in the drill hole before implant placement is an appealing approach.

**Aim:**

The aim of this study was to use short distance support immersion endoscopy (SD-SIE) to compare the quality of regenerated bone in healed postextraction sites, which are grafted with an in situ hardening *β*-TCP, against ungrafted sites, before implant placement. This assessment was based on microscopic bone analysis in combination with the blood vessel count.

**Method:**

13 spontaneously healed and 13 grafted postextraction sites in 3 men and 6 women, aged 26–83 years, were evaluated using SD-SIE after 4–6 months. SD-SIE was applied in drill holes before implant placement, and videos were taken from representative central buccal areas. The video recordings were analyzed using Image J software for (1) number of blood vessels per area (NBV), (2) relative area of vessels (VA), (3) relative area of mineralized bone (MBA), (4) relative area of unmineralized bone (UMBA), and (5) relative area of bone substitute (BSA).

**Results:**

The grafted sites showed more (1) NBV as well as (2) VA (8.6 ± 1.1; 2.03 ± 0.28%) than the ungrafted sites (2.5 ± 0.6; 1.18 ± 0.36%) (independent *t*-test; *p* < 0.05); (3) MBA and (4) UMBA were similar to those in the grafted sites (86.3 ± 2.2 %; 13.7 ± 2.2 %) and to the ungrafted sites (89.5 ± 3.7%; 10.5 ± 3.6%) (independent *t*-test; *p* > 0.05); and (5) BSA in the grafted sites was 18.2 ± 5.4%.

**Conclusion:**

SD-SIE is an interesting new approach for in situ assessment of bone quality and blood supply before implant placement. The regenerated bone in *β*-TCP grafted extraction sockets showed an increased vascularization compared to ungrafted sites providing a vital support for subsequent implant placement.

## 1. Introduction

Regeneration of alveolar bone by applying the technique of guided bone regeneration has been well documented in experiments and clinical investigation [1]. Bone substitutes for the support of local regeneration must be biocompatible and osteoconductive to prevent the material becoming encapsulated by fibrous tissue, leading to device failure and the need of a second intervention. From a clinical perspective, bone graft substitutes should be moldable during application, display a self-stabilizing and hardening potential, and form a stable, but still porous, scaffold in the defect. This approach eases the application and, at the same time, would greatly reduce the need for membranes to retain loose graft materials in the defect, resulting in a shortened and simplified surgical procedure [1]. Moldable microporous, synthetic calcium phosphate bone graft substitutes (*β*-TCP or *β*-BCP), coated with a micrometer thin layer of a degradable polymer, have been shown to be valuable materials for alveolar ridge regeneration as well as socket preservation [2, 3]. Porous calcium phosphates have been under intense investigation for more than 20 years and are widely used as bone graft substitutes. Nowadays, calcium phosphate (CaP) materials may generally vary in their rate of in vivo degradation, structure, and mechanical strength. The optimum porosity for bone should allow a vascular ingrowth and corresponding bone substitute resorption rate, since an ideal regenerative scenario would consist of a biomaterial resorption rate timed with new osseous tissue ingrowth [4].

The formation of an adequate vascular network is crucial for osteogenesis during both development and repair. Therefore, an effective bone graft for bone augmentation in the maxillofacial region shall support the formation of an adequate vascular network providing nutrition, osteoprogenitor cells, and growth factors to the site. Moreover, adequate perfusion is not only a prerequisite for bone regeneration but also for osseointegration for subsequent place dental implants [5]. Therefore, it is highly important to define the conditions for ideal bone regeneration and to investigate aspects of vascular supply of regenerated areas to improve the success of implant therapy.

Improvements of bone regeneration will rely on previous research of bone blood flow, to determine the right combination of scaffold, cell source, growth factors, and biomechanical conditions [6]. In clinical situations, as a response to bone injury, such as a fracture, robust angiogenesis occurs to relieve oxygen tension and transport osteoprogenitor cells for repair.

Vessel size and number can be robustly quantified ex vivo using histology or vascular casting. However, accurate quantification of in situ blood flow, particularly in bone, is more difficult in humans, when experimentally used techniques in animal trials such as radioactive microspheres cannot be applied.

There are two basic mechanisms of blood vessel formation: vasculogenesis (formation of a blood vessel from a progenitor cell, angioblast or hemangioblast) and angiogenesis (new vasculature development from the preexisting blood vessels). Additionally, a body of experimental work has shown that the presence or absence of the periosteum significantly affects the healing response [7].

For instance, Weigand et al. [8] hypothesized that extrinsic and intrinsic vascularization of a large-size bone tissue construct determines a successful regeneration process within a bone defect. The authors microsurgically created an arteriovenous loop and connected it with the bone substitute in either perforated titanium chambers (intrinsic/extrinsic) or tested the isolated Teflon chambers (intrinsic). In the intrinsic vascularization model, degradation of the scaffold and osteoclastic activity was significantly lower after 18 weeks, compared to the combined intrinsic-extrinsic model [8].

For observation of bone perfusion on a microscopic level, intravital microscopy has been used [9]. More recently, bone vascular parameters have been evaluated using an optical bone chamber implanted onto the calvaria of the rabbit [10]. For in vivo evaluation of soft tissues, light microscopy, in particular, epifluorescence and confocal microscopy, may be applied where the spatial resolution is typically limited to 50–100 microns of thickness of the tissue [11].

Based on earlier reports [9, 12, 13], and the refinement of the evaluation procedures, endoscopic tools have recently reached a similar magnification to the clinical observation of bone surfaces in vivo similar to the experimental setting. The short distance support immersion endoscopy (SD-SIE) allows to do an almost histological bone evaluation in vivo, not only under static, but also under dynamic conditions [12]. In this context, the field of dentistry provides a unique opportunity to get access to human bone surfaces during the preparation of implant sites prior to dental implants placement.

SD-SIE has been used as a diagnostic aid to judge the bone structure, to observe the presence of nutritional Volkmann's foramina of the alveolar bone [13]. Thus, it is possible to evaluate under high magnification, prior to implant placement, regenerated sites to detect new bone formation, to identify remnants of biomaterial, and to observe dynamic aspects of vascularization at specific sites.

The primary objective of this study was designed to evaluate the vascularization and, secondly, to microscopically evaluate the bone quality of postextractions sites grafted with a moldable microporous, synthetic beta tri-calcium phosphate (*β*-TCP) bone graft substitute, in comparison to ungrafted sites before implant placement after 4–6 months. This assessment was performed using SD-SIE.

### 1.1. Patients and Methods

**Figure 1:**
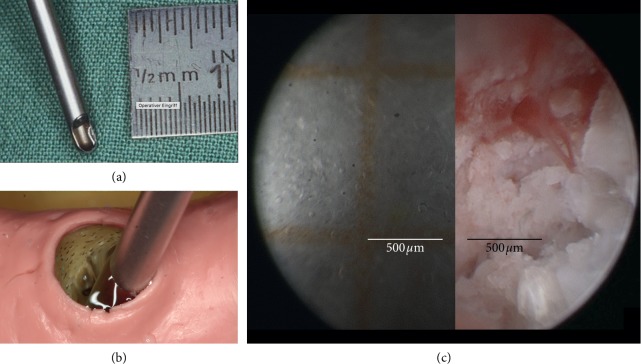
SD-SIE: (a) the endoscope is inserted into a sheath that provides both support and an irrigation system. (b) The endoscope with support immersion shaft is placed inside a dental extraction site model and implant cavity. (c) Short distance endoscopy allows observation under adequate magnification to differentiate structures for micromorphologic evaluation. When positioned at the entrance or fundus of the cavity, the endoscope can provide either a general or detailed view of the surgical field without having to make additional adjustments, thanks to its high depth of field.

A pilot prospective case control study was performed in the Implant Clinic of the Dental School of Universidad de la Frontera (UFRO), Temuco, Chile, between 2017 and 2018. A total of nine patients (3 men and 6 women, aged 26 to 83 years) agreed to participate in this study, and signed informed consents were completed at the study visit at the Implant Clinic of the Dental School of UFRO. The study was conducted in line with the principles outlined in the Declaration of Helsinki (2008) on experimentation involving human participants. Ethics approval for the conduct of the study was granted by the Universidad de La Frontera Ethics Committee (Decision 118/16), DIUFRO project No. DI17-0170. These participants were selected for ridge preservation of alveolar extraction sites with a moldable self-hardening synthetic bone graft substitute (GUIDOR easy-graft CLASSIC, Sunstar Suisse SA, Etoy, Switzerland). Socket grafting was carried out following tooth extraction according to the concept of ridge preservation to maintain the lateral contour of the alveolar ridge. A total of 13 grafted and 13 ungrafted alveolar sites were analyzed with SD-SIE in the drill hole right before implant placement (Figure 1). Details of the implant sites are given in Table 1. The sites belonged to the anterior maxillary zone including premolars, canines, and incisors. All participants' data were anonymized to ensure confidentiality and the grafted and ungrafted groups were randomized for a blind analysis.

### 1.2. Endoscopic Observation

For SD-SIE, a rigid STORZ endoscope with 1.9-mm diameter, 30 and 70° view angle and an integrated support-irrigation tube (Karl Storz, Tuttlingen, Germany) was used. The endoscopes were coupled to a Karl Storz Image 1 HD Camera 222200 50 (Karl Storz, Tuttlingen, Germany). Before the micromorphologic evaluation, the scope window was placed in contact with the surface of the graph paper and the scale was set to 500 micrometer (see Figures 1(a)–1(c)). The graph paper was placed inside the dental model filled with water. This model allowed us to proceed to the morphologic evaluation within a natural chamber formed by the implant cavity (see Figure 1(c)).

**Figure 2:**
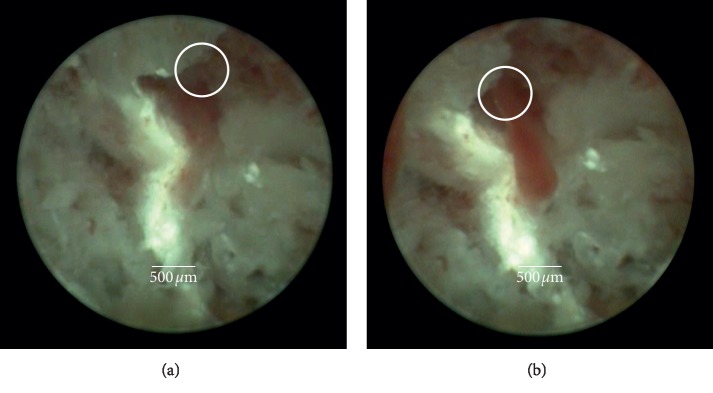
Observation of regenerated bone using SD-SIE: (a) region of interest showing initial bleeding of a blood vessel in regenerated bone. (b) During reduced rinsing pressure, extravasation of blood increases.

Videos were taken from the central buccal aspect of the drill hole. Manual jet stream irrigation was served to clean the bone surface, and the irrigation flow was stopped immediately before observation (Figure 2); thus, the bone surface could be observed without pollution through the irrigation medium (saline solution).

### 1.3. Image Selection

Taking the surgical reports as reference, the sites were identified and cross-checked for presence of bone substitute. The central buccal area of the augmented or native cavities was examined as a representative zone for blood vessel counts as well as microscopic bone imaging, and apical and cervical regions were not included in the evaluation. Screenshots were taken from the original digital video archives (mpg4 files) and stored in jpg format. The image analysis program (Image J, V.64, National Institutes of Health, Bethesda, MD, USA) was used to open digitized endoscopic images.

### 1.4. Vessels, Mineralized, and Unmineralized Bone Structure Analysis

**Figure 3:**
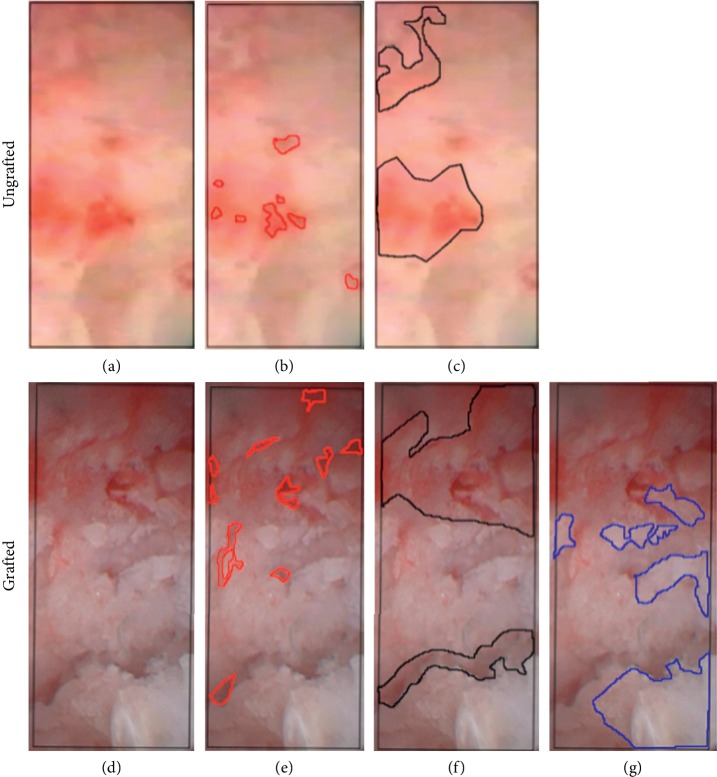
Micromorphology. Bone analysis of grafted and ungrafted bone: (a, d) Observation of bleeding vessels; (b, e) assessment of blood vessel area; (c, f) nonmineralized bone area; (g) grafted bone area.

The analysis of the bleeding bone surface was performed according to the following protocol: (1) selection of the area of interest (AoI), (2) screenshot of images after irrigation and cleaning, and (3) set of the scale bar according to the transversal diameter of the scope window (contact mode) at the center of the image and definition of the area of measurement using Image J (see Figure 1). The rectangular selection tool was used to determine the AoI with a scale of 500 micrometer and the bone structures from native ungrafted (*n* = 13) and grafted (*n* = 13) were selected from the freehand selection tool from Image J software (see Figure 3). Total number and area of vessels (red selection Figures 3(b) and 3(e)), nonmineralized bone (black selection Figures 3(c) and 3(f)), and easy-graft graft material in the bone substitute group (blue selection Figure 3(g)) were observed. Vessels were identified by observing the original video recordings that were recorded by the endoscopic procedure in vivo (Figures 3(a) and 3(d)) after cleaning the bone surface with saline solution. The percentages were calculated from area of mm^2^ by (unmineralized bone or vessels) multiplied by 100 and then divided by the AoI (Figures 3(a)or 3(d)). The mineralized bone area was calculated from the difference of the AoI from the vessels area selected (Figures 3(b) and 3(e)) plus nonmineralized bone (Figures 3(c) and 3(f)). The bright white areas were selected as bone substitute (Figure 3(g)). Vessels were counted in a blinded manner by three of the authors independently, for further processing, the numbers were averaged. Sites with pulsatile extravasation from arterial vessels were excluded, if the AoI could not be cleansed sufficiently.

### 1.5. Statistical Analysis

Shapiro–Wilk's test (*p* > 0.05) showed that the data are normally distributed for unmineralized and mineralized bone, total area, and number of total vessels. Therefore, the independent *t*-test was used to see the differences of structures between ungrafted and grafted sites. The level of significance was set at *p* < 0.05. Statistical analysis was performed using SPSS, Inc., software (version 23).

## 2. Results

SIE applied in ungrafted and grafted sites in situ allowed for the quantitative assessment of internal bone surface. Structures such as vascular canals can be identified under variable magnification. Microscopic bone imaging analysis based on SIE allowed the identification of the quality of the localized areas of internal bone surface by the relative assessment of mineralized and unmineralized zones and vascular canals. The grafted sites showed a higher number of NBV and a larger area (VA) of blood vessels (8.6 ± 1.1; 2.03 ± 0.28%) compared with the ungrafted sites (2.5 ± 0.6; 1.18 ± 0.36%), as shown in Tables 2 and 3. In relation to mineralized (MBA) and unmineralized (UMBA) bone areas, the grafted sites did not show differences of percentage areas (86.3 ± 2.2%; 13.7 ± 2.2%) with the ungrafted (89.5 ± 3.7%; 10.5 ± 3.6%) (Table 3) (independent *t*-test; *p* < 0.05). The grafted sites showed a relative area of bone substitute in a mean of 18.2 ± 5.4% (Table 3).

## 3. Discussion

The first in vivo observation of Volkmann's channels using SIE as a microscopic tool was described by Beltrán et al. [3]. Meanwhile, the assessment of the osseous morphology to determine vascular and structural bone quality has been defined as a quantitative analysis of relative area of vessels (VA), mineralized (MBA) and unmineralized bone area (UMBA), and relative area of bone substitute (BSA) in percentages (Table 3). Grafted sites showed differences in comparison to the ungrafted sites (independent *t*-test; ^*∗*^*p* < 0.05).

Engelke et al. [12] reported on microscopic bone analysis (MBA) using a SD-SIE based protocol with subsequent Image J analysis. MBA allowed a quantitative description of bone morphology such as unmineralized and mineralized areas as well as area of vascular canals under bleeding in vivo condition. The morphometric examination revealed that there was more unmineralized bone and less vascular canal area in implant sites than in the extraction sockets.

The present study was focused on the vascular supply in grafted vs. ungrafted postextraction sites, i.e., on the quantification of blood vessels per area. The study was conducted in patients who required ridge preservation with bone graft substitute following tooth extraction. Grafting of postextraction sites was performed according to the manufacturer's instruction following tooth extraction with various degrees of alveolar bone loss in the sense of ridge preservation. Since there was no primary protocol to assess the bone loss, the observation was focused on the typical area graft which is located at the buccal wall. The central buccal area was chosen in order to avoid possible influence of occlusal loading in the crestal zone or missing augmentation in the apical area. By previous observation of resting bone substitute particles, it was confirmed that the AoI corresponded to the augmented zone.

Methodically, the blood vessel count is limited by the conditions of the observation, in particular, the resolution of the optical system. The resolution of in vivo light microscopy for soft tissues using epifluorescence and confocal microscopy is typically limited to the outer 50–100 microns of the accessible tissue. With reference to Engelke et al. [12], the canal diameter of Volkmann's canals lies between 30 and 50 microns; these canals are clearly visible and confirm that the resolution of SIE is well above the average diameter of cortical canals. For the purpose of the present study, a resolution of 50 microns was assumed.

There are no in vivo data available on the number of blood vessels in alveolar bone. However, the literature offers a wide range of numbers of blood vessels per area in different bone structures, tissues, and animal models.

Sezer et al. [13] reported on bone marrow microvessel density (MVD) in immunohistochemical CD34 stained paraffin-embedded bone marrow biopsies and survival in patients with multiple myeloma. The median MVD was 48 vessels/mm^2^; the range was 0–125 vessels/mm^2^. The regenerated alveolar bone, however, does not represent pure bone marrow conditions as used in the clinical investigation mentioned.

There is no methodological consensus on the parameter, nomenclature and regions of interest for the quantification of bone marrow vessels. This leads to a pronounced heterogeneity of the results among the various publications.

Bone marrow vessels are heterogeneous and include small classic capillaries (10–15 *μ*m) and a majority of sinusoid capillaries (20–30 *μ*m), which belong to the venous capillary system [14]. These vessels probably are prone to be disregarded during a SD-SIE evaluation due to their small diameter and due to missing staining in vivo. According to our previous evaluation, these capillary structures are at the limit of the presently available resolution of SD-endoscopes of 50 *μ*m. A comparable magnitude of vessels per tissue area were reported by Hettrich et al. [15] who evaluated the effect of rhPTH on the healing of tendon to bone in a rat model. Factor VIII staining was used to evaluate angiogenesis within the supraspinatus tendon and tendon-bone interface. Microscope field images at 40x magnification were centered over the area of insertion, the vessels within these areas were counted, and the number of vessels per mm^2^ was calculated. The blood count figures ranged between 0 and 3.5 vessels per mm^2^, i.e., the condition of the tendon insertion appears to lie in the same range as our observations in alveolar bone.

For clinical purposes, it is highly important to define the conditions for ideal bone regeneration after tooth extraction and to investigate aspects of vascular supply of regenerated areas to improve the success of implant therapy.

Weiss et al. [16] observed the blood vessel formation in Collagen HA (CHA) scaffolds with occlusive membrane (OM) implanted in a mouse model and evaluated with *μ*CT :CHA OM yielded the most blood vessels (8.22 ± 1.19) per unit area (0.3588 mm^2^), followed by Col OM (6.61 ± 0.64) and CHA acellular (6.00 ± 0.80) groups. In our in vivo observations, the grafted areas showed more vascularization (8.6 ± 1.1) per AoI than the ungrafted sites (2.5 ± 0.6) similar to previous studies in animal models. Thus, we may conclude that regenerated bone at a comparable regeneration time following extraction appears to increase the numbers of vessels.

The present in vivo observation exhibited that following the grafting of postextraction sites with a moldable in situ hardening *β*-TCP, a direct contact of vessels with the TCP regularly occurs. The material was in full contact with the adjacent capillaries without interposition of connective tissue or avascular zones. This may be taken as an indicator for an adequate regeneration and maturation of the sites. The findings are in accordance with reports of Valdivia-Gandur et al. [17] and Schmidlin et al. [1] who described a good biocompatibility of this bone graft substitute with an intimate distribution of newly formed bone and bone graft substitute materials.

Endomicroscopy of bone as proposed in the actual study design also allows to determine blood flow parameters and a variety of bone pathologies in vivo. For clinical purposes, it is highly important to define the conditions for ideal bone regeneration after tooth extraction and to investigate aspects of vascular supply of regenerated areas to improve the success of implant therapy.

Evaluation for bone reconstruction should focus on three levels of graft integration with surrounding tissues: osseointegration, vascular integration, and gradual substitution by bone remodeling under functional loading to achieve complete integration [18]. Osteogenic and vascular regeneration need to be complementary and should proceed at satisfactory rates for effective repair and functional restoration. The present study confirms that a similar range of vascular supply takes place within the sites grafted with in situ hardening moldable bone substitute compared to ungrafted sites. Degradation of TCP was observed close to the defect borders after 16 weeks where the formerly round granules were integrated in bone and displayed irregular shapes [1]. This could also be confirmed by nonquantified observation in vivo, where the limit of the implant cavity was located directly adjacent to the buccal border of the alveolus.

In animal trials, the number of penetrating vessels into a capsule with different bone regeneration constructs in the mouse model [19] was taken as an indicator for successful integration of constructs in vivo; however, the biomechanical conditions were significantly different from the site of implantation of the scaffold structure. Keeping this in mind, the present study provides conditions for a huge number of investigations to judge the outcome of bone regeneration constructs directly in situ before charging them with implants on a regular basis. Because there is no need for biopsies, endomicroscopy can be ethically justified due to the diagnostic value of the nondestructive evaluation of bone structure. It may deliver important parameters which could previously be acquired only by conventional evaluation of histological specimens under experimental conditions.

A precise definition of the area of observation within the clinical situs is mandatory, which can be documented directly and compared with previous 3D imaging technologies. It makes way for future outline micromorphological evaluation of regenerated sites and contributes to a newdata base for successful use of grafted alveolar sites.

However, there are some shortcomings for the method, which are mainly related to the limited image resolution. Without the use of an HD camera, the detection of capillary vessels may be impossible. Strong bleeding still impedes a clear surface observation. Furthermore, there is still a distortion of the view mainly in the vertical axis of the bone cavity and irregularities of the surface observed angle, if the endoscope cannot be placed in full contact with the surface irregularities.

## 4. Conclusion

SD-SIE allows for a morphometric in situ evaluation and quantification of the vascularization of human internal osseous surfaces after bone regeneration at the microscopic level. Therefore, it represents an interesting new approach for in situ assessment of bone quality and blood supply before implant placement. The regenerated bone in *β*-TCP grafted extraction sockets showed increased vascularization compared to ungrafted sites providing a vital support for subsequent implant placement.

## Figures and Tables

**Table 1:** Distribution of implant sites. Second premolar (PM2), first premolar (PM1), canine (C), lateral incisive (LI), and central incisive (CI).

SITE	PM2	PM1	C	LI	CI
Ungrafted sites	3	4	2	1	4
Grafted sites	2	4	3	–	3

**Table 2:** Quantitative analysis of number of blood vessels per area of interest (AoI) (NBV). Grafted sites showed more total number of vessels than ungrafted sites (independent *t*-test; ^*∗*^*p* < 0.05).

	Ungrafted sites (*n* = 13)	Grafted sites (*n* = 13)
NBV		
Mean	2.538	^*∗*^8.615
SD	2.259	4.011
SE	0.652	1.158
Min-max	0–6	1–14

**Table 3:** Quantitative analysis of relative area of vessels (VA), mineralized (MBA) and unmineralized bone area (UMBA) and relative area of bone substitute (BSA) in percentage. Grafted sites showed differences in compare the ungrafted sites (independent *t*-test; ^*∗*^*p* < 0.05).

	Ungrafted sites (*n* = 13)	Grafted sites (*n* = 13)
VA		
Mean	1.82	^*∗*^2.030
SD	1.274	0.967
SE	0.368	0.279
Min-max	0–4.34	0.28–3.53

MBA		
Mean	89.509	86.282
SD	12.77	7.876
SE	3.686	2.274
Min-max	60–100	62–95

UMBA		
Mean	10.491	13.718
SD	12.77	7.876
SE	3.686	2.274
Min-max	0–40	5–37

BSA		
Mean	0	18.199
SD	0	18.72
SE	0	5.404
Min-max	0	2–74

## Data Availability

Data that support the conclusions of this article are completely included in the manuscript.
